# Pioglitazone, a PPAR-y agonist, as one of the new therapeutic candidates for C3 glomerulopathy

**DOI:** 10.1007/s00467-023-06088-5

**Published:** 2023-07-26

**Authors:** Elia Balestra, Egidio Barbi, Viola Ceconi, Vittorio Di Maso, Ester Conversano, Marco Pennesi

**Affiliations:** 1 Department of Medicine, Surgery, and Health Sciences, University of Trieste, Trieste, Italy; 2 Paediatric Department, Institute for Maternal and Child Health-IRCCS “Burlo Garofolo”, Trieste, Italy; 3 Department of Nephrology, Cattinara Hospital, Azienda Sanitaria Universitaria Giuliano-Isontina (ASUGI), Trieste, Italy

**Keywords:** C3-glomerulopathy, Steroid-resistant nephrotic syndrome, Pioglitazone, Proteinuria, Children

## Abstract

**Background:**

C3-glomerulopathy (C3G) is a rare pediatric kidney disease characterised by dysregulation of the alternative complement pathway, with glomerular deposition of C3. C3G may often present as a steroid-resistant nephrotic syndrome (SRNS), and there is no established effective therapy: the usual treatment involves corticosteroids and immunosuppressive drugs. Pioglitazone, a PPAR-γ agonist with a protective action on podocytes, was reported in a few cases as helpful in reducing proteinuria when combined with steroids.

**Case-Diagnosis/Treatment:**

We report the case of a 13-year-old girl with silent past medical history who presented with SRNS. A kidney biopsy showed findings indicative of C3G. A low sodium diet and angiotensin-converting enzyme inhibitor were started; immunosuppressive treatment with mycophenolate mofetil (MMF) was administered due to the cortico-resistance. Because of poor response to the immunosuppressant, a trial with eculizumab was attempted without significant response and persistence of proteinuria in the nephrotic range. A further therapeutic trial was performed with tacrolimus with no disease remission. Due to a severe deterioration in her condition, the girl was hospitalized and treated with high-dose steroid bolus. A daily dose of oral prednisone and MMF were re-started without benefit with persistent levels of nephrotic range proteinuria. The administration of pioglitazone consistently lowered proteinuria levels for the first time since the onset of the disease, with a maintenance of the effect and normalization (< 0.15 g/24 h) at the 10-month follow-up.

**Conclusions:**

In this patient affected by C3G, pioglitazone proved effective in reducing proteinuria levels.

## Introduction

Nephrotic syndrome (NS) is childhood's most frequent pediatric glomerular disease, with 2–7 cases/100,000 incidences [1]. It is characterized by nephrotic-range proteinuria (urinary protein/creatinine ratio ≥ 2 mg/mg, or ≥ 1000 mg/m^2^ per day), hypoalbuminaemia (< 30 g/L) and oedema [2].

Oral steroids effectively induce remission within 4–6 weeks of steroid course in 75–90% of NS cases, with the remaining 10–25% being unresponsive to the treatment, hence classifying as steroid-resistant nephrotic syndrome (SRNS). High doses of intravenous steroids can also be considered for severe disease. SRNS can be genetic or immune-mediated and is characterised by worse prognosis [3, 4], with up to 30% of SRNS also being resistant to immunosuppressive therapies [5].

C3 glomerulopathy (C3G) is a rare kidney disease (0.2–1.0 cases per 1,000,000 in Europe) which can present as SRNS. It is caused by dysregulation of the complement cascade [6], especially in the alternative pathway, resulting in a build-up of C3 and its cleavage products in the glomerular tissue, causing inflammation and progressive kidney disease. Depending on the location and characteristics of deposits, C3G is subdivided into two disease forms, with some overlapping clinical and pathological features: dense deposit disease (DDD) and C3 glomerulonephritis (C3GN) [4].

Dysregulation and hyperactivation of the complement cascade represent the central point in the pathogenesis of the disease. It can result from genetic mutations in complement genes (i.e. mutations of C3 that enhance C3 convertase or mutations of factor H) or from the presence of auto-antibodies against complement components (i.e. antibodies stabilizing the C3 convertase or against factor H/factor H receptor which enhance C3 convertase activity). Genetic mutations are found in up to 30% of the patients presenting C3G, mainly affecting the *C3* gene, *CFB*, *CFH*, *CFI* and *CFHR* [7, 8].

The primary autoantibodies involved in C3G are the so-called nephritic factors (NeFs), which stabilize C3 or C5 convertases and prolong their half-life. Other autoantibodies include anti-FH, anti-FB, and anti-C3b [8].

C3G can have various clinical manifestations, ranging from mild proteinuria and microscopic haematuria with normal kidney function and no clinical sign of disease to nephrotic syndrome, gross haematuria, hypertension and renal insufficiency (serum creatinine elevation) [7].

Characteristic laboratory findings are low levels of C3. An infection can precede the onset of the disease, and C3G should be suspected instead of post-infectious GN when low serum C3 levels persist beyond 8–12 weeks [8].

Optimal treatment for C3G still represents a medical challenge, and no broadly validated evidence exists to guide therapeutic choice. Standard care is blood pressure control, which should be optimized with a low-salt diet and anti-proteinuric drugs such as angiotensin-converting enzyme inhibitors (ACEi) or angiotensin II receptor blockers (ARB).

In case of moderate to severe disease, as in patients presenting with NS, it is suggested to start with high doses of oral glucocorticoids for 4–6 weeks (60 mg/m^2^), followed by gradual tapering. Combination therapy with mycophenolate mofetil (MMF) showed a particular efficacy in achieving remission in the case of persistent proteinuria.

Complement-targeted therapy with eculizumab or other terminal complement pathway blockers was effective in some patients. Rituximab and plasma exchange were studied in C3G; however, substantial results supporting their use still need to be provided. However, many patients have multidrug-resistant glomerulopathy, and recurrence is widespread in those who eventually undergo a kidney transplant [6, 7].

The long-term outcome for C3G is poor kidney function, particularly in patients presenting with nephrotic syndrome, renal insufficiency, and crescents on initial biopsy; up to 30% of pediatric patients evolve to chronic renal insufficiency [9, 10].

PPAR-γ (peroxisome proliferator-activated receptor gamma) agonists, such as pioglitazone, were initially introduced to treat insulin resistance in type II diabetes mellitus, showing the ability to reduce albuminuria. Recently, some studies have highlighted an additional potential role in reducing proteinuria in non-diabetic kidney diseases [11, 12]. At the base of their effectiveness, there would be direct podocyte protective mechanisms, such as an enhanced expression of structural glomerular proteins and a reduction of injury-induced podocyte apoptosis [11].

We report the case of an adolescent girl affected by C3 glomerulopathy presenting as SRNS, with currently available treatments unable to induce remission; the disease showed additional improvement in levels of proteinuria and maintenance of remission, other than an overall immunosuppression reduction, after the introduction of pioglitazone concomitant therapy.

## Case presentation

A 13-year-old girl (body weight 51.1 kg, height 158 cm) was admitted with nephrotic syndrome (urinary protein 7.19 g/24 h), hypertension and microscopic haematuria. Laboratory findings revealed low serum albumin (1.9 g/dL) and C3 levels (C3 25 mg/dL, n.v. 85–142 mg/dL; C4 20 mg/dL, n.v. 12–41 mg/dL), dyslipidaemia; ANA, ANCA and anti-DNA tested negative (Table 1).

**Table 1:** Laboratory and genetic test results at disease onset

Laboratory test results	Bioptic sample
• C3 25 mg/dL n.v. 85–142 mg/dL; C4 20 mg/dL, n.v. 12–41 mg/dL • ESR, CRP: negative • ANA, ANCA, anti-DNA antibodies: negative • Autoantibodies anti-FH: 11.45 AU/mL (n.v. < 56 AU/mL) • IgG 438 mg/dL, IgA 194 mg/dL, IgM 102 mg/dL • sC5b-9: 1208 ng/mL	• Light microscopy: diffusely pathological glomeruli, significant mesangial hyperproliferation and hypercellularity. GBM thickened and duplicated. Normal tubules, interstitium and arteries • Electron microscopy: thickened and split GBM. Extensive fusion of the pedicels and marked villous hyperplasia of the podocytes. Endothelium with normal fenestration. Areas of mesangial expansion with hypercellularity. No evidence of DDD • Immunofluorescence: C3 + + + + , C1q/IgM + +
Genetic analysis • Homozygous polymorphism p.V62I for *CFH* gene—associated with C3G • Homozygous polymorphism C.*897 T > C for *MCP* gene—associated with aHUS	

*aHUS*, atypical haemolytic uremic syndrome; *CRP*, C reactive protein; *C3G, *C3 glomerulopathy; *DDD*, dense deposit disease; *ESR*, erythrocyte sedimentation rate; *GBM*, glomerular basement membrane; *n.v.*, normal values

Oral steroid treatment was started at 60 mg/m^2^/day, but the disease proved to be cortico-resistant. For this reason and age criterion, a kidney biopsy was performed. The procedure indicated the presence of membranoproliferative glomerulonephritis (MPGN) with predominant C3 complexes and limited immunoglobulin deposits, indicative of C3 glomerulopathy (C3G). Next Generation Sequencing (NGS) panels did not reveal pathogenic mutations, but genetic variants of *CFH* and *MCP* genes associated with a higher risk of the disease (homozygous polymorphism p.V62I for *CFH* – associated with C3G – and homozygous polymorphism C.*897 T > C for *MCP* – associated with the atypical haemolytic uremic syndrome (aHUS)). Anti-H factor autoantibodies (11.45 AU/mL with average values < 56 AU/mL) and C3 nephritic factor resulted within the normal range; the high serum levels of C5b-9 (1208 ng/mL) were indicative of complement activation (Table 1).

Supportive treatment with a low sodium diet and nephron-protective therapy with ramipril 5 mg × 2/day was started. Because of the cortico-resistance displayed by the NS, MMF was introduced, whose dosage was progressively optimised (up to 1250 mg × 2/day, approximately 1250–1500 mg/m^2^/day) and monitored through target drug monitoring (TDM), while prednisone was slowly tapered in the following months due to its lack of efficacy and potential adverse effects.

Due to the poor response to MMF, an off-label trial with the terminal complement blocker eculizumab was attempted nearly 2 years after disease onset to inhibit complement pathway dysregulation. For 6 months, the girl underwent drug injection every two weeks: despite achieving a normalisation of C5b-9 levels in laboratory tests (324 ng/mL after 3 months of treatment), the therapeutic course produced no clinical benefits and the nephrotic-range proteinuria persisted.

Eculizumab administration was therefore suspended and a further therapeutic trial was performed with tacrolimus (0.15 mg/kg/day), a calcineurin inhibitor, without achieving disease remission and observing a mild transient reduction of kidney function (urea 65 mg/dL, creatinine 1.38 mg/dL, eGFR by Schwartz 66 mL/min/1.73 m^2^) instead during its administration.

After about two and a half years since the onset of the disease, the girl was re-admitted due to a critical worsening of her condition, with a peak proteinuria of 15.26 g/24 h, peripheral oedema and hypertension. The patient was treated with high-dose steroidal bolus, followed by the reintroduction of oral prednisone daily dose. Due to its substantial ineffectiveness and potential nephrotoxicity, tacrolimus administration was suspended, and MMF re-started.

**Fig. 1:**
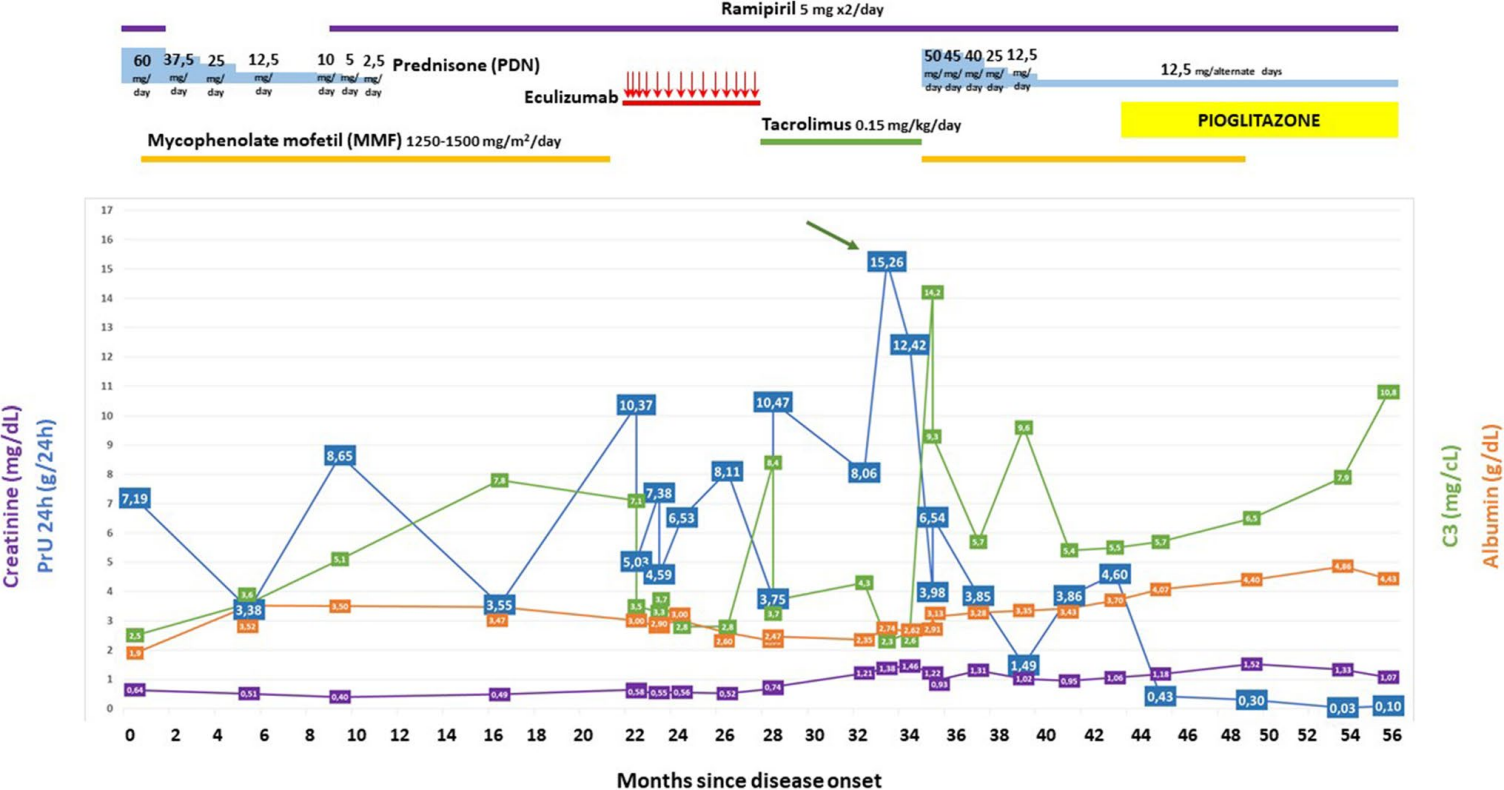
Graph showing the trend over time of 24-h proteinuria levels. Green arrow: High-dose steroidal bolus during hospital re-admission. Red arrows: Multiple eculizumab doses. C3 levels must be multiplied by 10 to be expressed in mg/dL as in the text

In the few months following discharge, clinical signs of the disease (i.e. oedema, infections) were absent, and proteinuria levels were temporarily reduced. However, normalisation was never achieved (best value of 1.49 g/24 h), and the trend worsened over time (Fig. 1). Due to adverse effects, such as weight gain and a worsening perception by the patient of her body image, steroid therapy was progressively reduced. At the same time, MMF dosage was constantly monitored and adjusted with TDM. Laboratory tests showed normal creatinine levels and kidney function in range.

Considering the persistent levels of nephrotic-range proteinuria, a trial with pioglitazone was started at the daily oral dose of 30 mg/day. Forty days later, proteinuria levels were significantly reduced (0.43 g/24 h) for the first time since the disease onset (Fig. 1). Four months following the beginning of the therapy, proteinuria levels were consistently low (0.30 g/24 h), leading to the tapering of MMF while maintaining the minimal dose of PDN, according to the recent evidence of the synergistic effect of co-treatment with PPAR-γ and glucocorticoids. Another four and six months later, proteinuria levels were in range (respectively 0.03 g/24 h and 0.1 g/24 h) (Fig. 1). Considering the associated average blood pressure values, a gradual dosage reduction of ACEi was attempted but eventually stopped in light of a mild trend of proteinuria increase. Together with that, C3 levels improved (C3 108 mg/dL) after pioglitazone introduction (Fig. 1). During pioglitazone administration there was a transient worsening of kidney function (creatinine 1.52 mg/dL, eGFR by Schwartz 60.1 mL/min/1.73 m^2^), which improved over time (creatinine 1.07 mg/dL, eGFR by Schwartz 85.3 mL/min/1.73 m^2^ after ten months of treatment) without dose adjustment. All the proteinuria values were measured through 24-h urine collections and analyzed in the same laboratory of our hospital. Proteinuria levels over 24 h were considered normal if ≤ 0.1 g/m^2^/day (our girl presented a body surface area equal to 1.6). No side effects or problems of therapeutic compliance were reported.

## Discussion

The evidence to guide the choice of therapy in C3G is still limited.

In this case, all the pharmacological therapeutic options were attempted without achieving clinical remission of the disease. Otherwise, pioglitazone reduced proteinuria and its potential synergic effect with steroids allowed the discontinuation of MMF, hence minimising immunosuppression (Table 2) [13, 14]. We also speculate that pioglitazone reduced disease activity, as demonstrated by improved C3 levels after its administration.

**Table 2:** Overall immunosuppression after pioglitazone introduction

1 month before pioglitazone introduction (43 months since disease onset)	1–4 months after pioglitazone introduction (45 and 48 months since disease onset)	8 months after pioglitazone introduction (52 months since disease onset)	10 months after pioglitazone introduction (54 months since disease onset)
Immunosuppressive treatment: MMF 1000 mg × 2 /day—1.219 mg/m^2^/day (score 4) Prednisone 12.5 mg/alternate days—3.81 mg/m^2^/day (score 1)	Immunosuppressive treatment: MMF 1000 mg × 2 /day—1.219 mg/m^2^/day (score 4) Prednisone 12.5 mg/alternate days—3.81 mg/m^2^/day (score 1)	Immunosuppressive treatment: Prednisone 12.5 mg/alternate days—3.81 mg/m^2^/day (score 1)	Immunosuppressive treatment: Prednisone 12.5 mg/alternate days—3.74 mg/m^2^/day (score 1)
TIS = 4 + 1 = 5	TIS = 4 + 1 = 5	TIS = 1	TIS = 1

Total Immunosuppression Score (TIS) is a score used to make objectifiable the overall immunosuppressive treatment at varying doses over time [13, 14]. For each class of immunosuppressive drug administered, a score from 0 to 4 is assigned depending on the percentage of a full dose for patient’s body size (0 = 0%; 1 =  < 25%; 2 =  ≥ 25– < 50%; 3 =  ≥ 50– < 75%; 4 =  ≥ 75%)

Therefore, when facing steroid- and immunosuppressant-resistant NS, a new therapeutic option may be offered by thiazolidinediones (TZDs), such as pioglitazone.

TZDs are PPAR-γ agonists, introduced initially to treat type II diabetes mellitus; they have been demonstrated to reduce albuminuria in type 2 diabetes [15], as well as to decrease proteinuria in non-diabetic kidney diseases [16].

In preclinical studies, TZDs were demonstrated to protect podocytes directly through different mechanisms, such as enhancing the expression of podocyte-specific proteins contributing to the structural maintenance of the glomerular barrier [11] and reducing injury-induced podocyte apoptosis. These effects are mediated by augmented cyclin-dependent kinase inhibitor p27 and the anti-apoptotic molecule Bcl-xL, with a significant decrease of pro-apoptotic caspase-3 activity [12].

Co-treatment with pioglitazone and glucocorticoids demonstrated a synergistic effect, with TZDs enhancing the effect of glucocorticoids in reducing proteinuria. A recent study by Agrawal et al. hypothesized crosstalk between their signaling pathways [13], with both drugs binding to their nuclear receptor belonging to the nuclear hormone receptor superfamily. Both drugs enhance glomerular synaptopodin and nephrin expression and reduce COX-2 expression after glomerular injury.

In clinical studies, TZDs effectively reduced proteinuria levels and systolic blood pressure in overweight adults with chronic non-diabetic kidney disease [17]. At the same time, a recent case series by Hunley et al. reported the efficacy and safety of adding pioglitazone to enhance proteinuria reduction in 8 children with idiopathic NS or primary focal segmental glomerulosclerosis (FSGS) [14].

In adult studies, the main adverse effects reported for pioglitazone are represented by the risk of cardiac dysfunction, weight gain, fluid retention, fractures and a controversial association with hepatic cancer. There are no data about the pediatric population. In our case, even if administrated only for 10 months, there was no evidence of short-time side effects (i.e. no oedema, steady patient weight). The transient and episodic worsening of kidney function during pioglitazone administration did not persist over time; moreover, considering the adolescent age of the patient, the absolute increase in creatinine levels was quite limited. While the reason of the impairment remains unclear, we speculate not to consider this as an adverse effect of pioglitazone (not reported in the current literature) or a demonstration of its lack of efficacy, considering the eventual rapid and persistent normalization over time.

## Conclusion

Although further confirmatory studies are needed, this report suggests that pioglitazone may represent a safe, non-immunosuppressive therapeutic option to reduce proteinuria in C3G with persistent and resistant proteinuria as well as, potentially, in other paediatric NS resistant to available therapies.

## Data Availability

The data that support the findings of this study are available from the corresponding (E.B.) author on request.

## Abbreviations

ACEi: Angiotensin-converting enzyme inhibitor
ARB: Angiotensin II receptor blocker
C3G: C3-glomerulopathy
C3GN: C3 glomerulonephritis
DDD: Dense deposit disease
FSGS: Focal segmental glomerulosclerosis
MPGN: Membranoproliferative glomerulonephritis
NGS: Next Generation Sequencing
NS: Nephrotic syndrome
PPARγ: Proliferator-activated receptor gamma
SRNS: Steroid-resistant nephrotic syndrome
TDM: Target drug monitoring
TZDs: Thiazolidinediones
